# People with epilepsy have poor life satisfaction and self-rated health: Findings from the United Kingdom

**DOI:** 10.3389/fpsyg.2022.986520

**Published:** 2023-01-17

**Authors:** Weixi Kang

**Affiliations:** Imperial College London, London, United Kingdom

**Keywords:** epilepsy, life satisfaction, SRH, self-rated health, wellbeing

## Abstract

Epilepsy is a neurological disorder characterized by brief, recurrent disturbances in the normal electrical functions of the brain that result in seizures. Although epilepsy is closely related to wellbeing, much less is known about how life satisfaction and SRH are affected by epilepsy in a nationally representative sample from the United Kingdom. The current research aims to investigate the difference in life satisfaction and SRH between people with epilepsy and people without epilepsy by using an innovative train-and-test approach on data collected between 2009 and 2010 from 428 people with epilepsy and 39,024 healthy controls while taking demographics into account. The results showed that people with epilepsy have both poorer life satisfaction and SRH compared to the scores that would be predicted by their demographics. This study implies that both life satisfaction and SRH are valid measures of wellbeing in people with epilepsy. Health professionals may utilize findings from the current study to come up with ways that can benefit wellbeing of people with epilepsy.

## Introduction

People with epilepsy, especially those with active seizures report worse quality of life satisfaction (defined as the subjective evaluation of one’s quality of life) than people without the disorders according to several previous studies (Kobau et al., 2008, 2012; Leidy et al., 1999; Boling et al., 2018). Moreover, people with epilepsy also face a lot of challenges in daily life including lower levels of education, reduced household income, unemployment, poor health, more disability, and more risky behaviors (Leidy et al., 1999; Centers for Disease Control and Prevention, 2005; Kobau et al., 2008, 2012; Layne Moore et al., 2009; Trinka et al., 2019). People with epilepsy are also more likely to perceive limitations in social and emotional support, which along with dissatisfaction with other aspects of life can lead to a decrease in overall life satisfaction (Kobau et al., 2008; Malvaso and Kang, 2022). Besides the above-mentioned psychological factors, neurological factors have adverse impacts on the quality of life in people with epilepsy (Livneh et al., 2001; Hermann and Jacoby, 2009) as well. While commonly used quality-of-life instruments for epilepsy have been applied in clinical settings and formally document patient concerns (Gilliam et al., 1997; Cramer et al., 1998; Martin et al., 2005; Yogarajah and Mula, 2019), much less is known about the relationship of epilepsy and life satisfaction in community-dwelling adults.

On the other hand, self-rated health (SRH) refers to one’s subjective assessment of health that integrates biological, functional, mental, and social aspects of a person (Wuorela et al., 2020), which include individual and cultural beliefs and health behaviors (Kobau et al., 2008). Although non-specific, SRH has good predictive validity such as being a strong predictor of mortality (Jylhä, 2009). There are few studies that have looked at SRH in people with epilepsy. For instance, Kobau et al. (2007, 2008) found that adults with active epilepsy and with a history of epilepsy were more likely to report fair or poor health in the United States.

Thus, although there are some studies that have examined the associations between epilepsy and life satisfaction, much less is known about how epilepsy is related to SRH given that SRH is related to various outcomes. In addition, most previous studies focused on small clinical samples, so there is a need for study at a large scale in community-dwelling adults. Moreover, most of these studies focused on context outside of the United Kingdom. Thus, the aim of the current study is to examine the relationship between epilepsy and life satisfaction and SRH using an innovative train-and-test approach that controls sociodemographic characteristics.

## Methods

### Data

This study extracted data from Understanding Society: the United Kingdom Household Longitudinal Study (UKHLS), which has been collecting annual information from the original sample of UK households since 1991 [when it was previously known as The British Household Panel Study (BHPS)]. Data were used from Wave 1, which was collected between 2009 and 2010 (University of Essex, Institute for Social and Economic Research, 2022). There were 428 people with epilepsy and 39,024 healthy controls in my study. Descriptive statistics can be found in Table 1.

**Table 1:** Descriptive statistics of demographic characteristics, life satisfaction, and SRH in healthy controls and people with epilepsy.

	**Healthy controls**		**People with epilepsy**	
	Mean	S.D.	Mean	S.D.
Age	45.78	17.96	43.16	15.94
Monthly income	1252.48	1354.79	1044.17	835.51
Life satisfaction	5.26	1.46	4.7	1.65
SRH	3.43	1.13	2.65	1.19
	N	%	N	%
**Sex**				
Male	17,096	43.81	178	41.59
Female	21,928	56.19	250	58.41
**Highest educational qualification**				
College	11,308	28.98	84	19.63
Below college	27,716	71.02	26,353	80.367
**Legal marital status**				
Single	19,057	48.83	241	56.31
Married	19,967	51.17	187	43.69

## Measures

### Epilepsy

Self-reported epilepsy is a valid measure to identify epilepsy at a population level (e.g., Brooks et al., 2012). Participants answered the question “Has a doctor or other health professional ever told you that you have any of these conditions? Epilepsy.” to indicate if they have epilepsy.

### Life satisfaction

Participants answered the question “How dissatisfied or satisfied are you with… your life overall?” using a 7-point scale ranging from 1 (not satisfied at all) to 7 (completely satisfied). The results of single-item measures and multi-item measures such as the Satisfaction with Life Scale (SWLS) have been shown to be very similar (Cheung and Lucas, 2014).

### SRH

Participants responded to the question, “In general, would you say your health is…” using a 5-point scale ranging from 1 (excellent) to 5 (very poor). The reliability of this single measurement of subjective health is moderate (e.g., Zajacova and Dowd, 2011). SRH was reverse coded, so now 1 = very poor and 5 = excellent.

### Demographics variable

Demographic variables include age, sex (male vs. female), monthly income, highest educational qualification (college vs. under college), and marital status (married vs. not current married).

### Analysis

An innovative train-and-test approach was used to analyze the current dataset, which is more advantageous than a paired sample *t*-test as it can control demographic covariates. First, two generalized linear models were applied by taking demographics as the predictors and life satisfaction and SRH as the predicted variables, respectively. Then, these models were used to predict the expected scores in people with epilepsy based on their demographic characteristics. Finally, two one-sample *t*-tests used to determine the difference between the actual scores in people with epilepsy. All analyses were performed on MATLAB 2018a.

### Results

**Figure 1:**
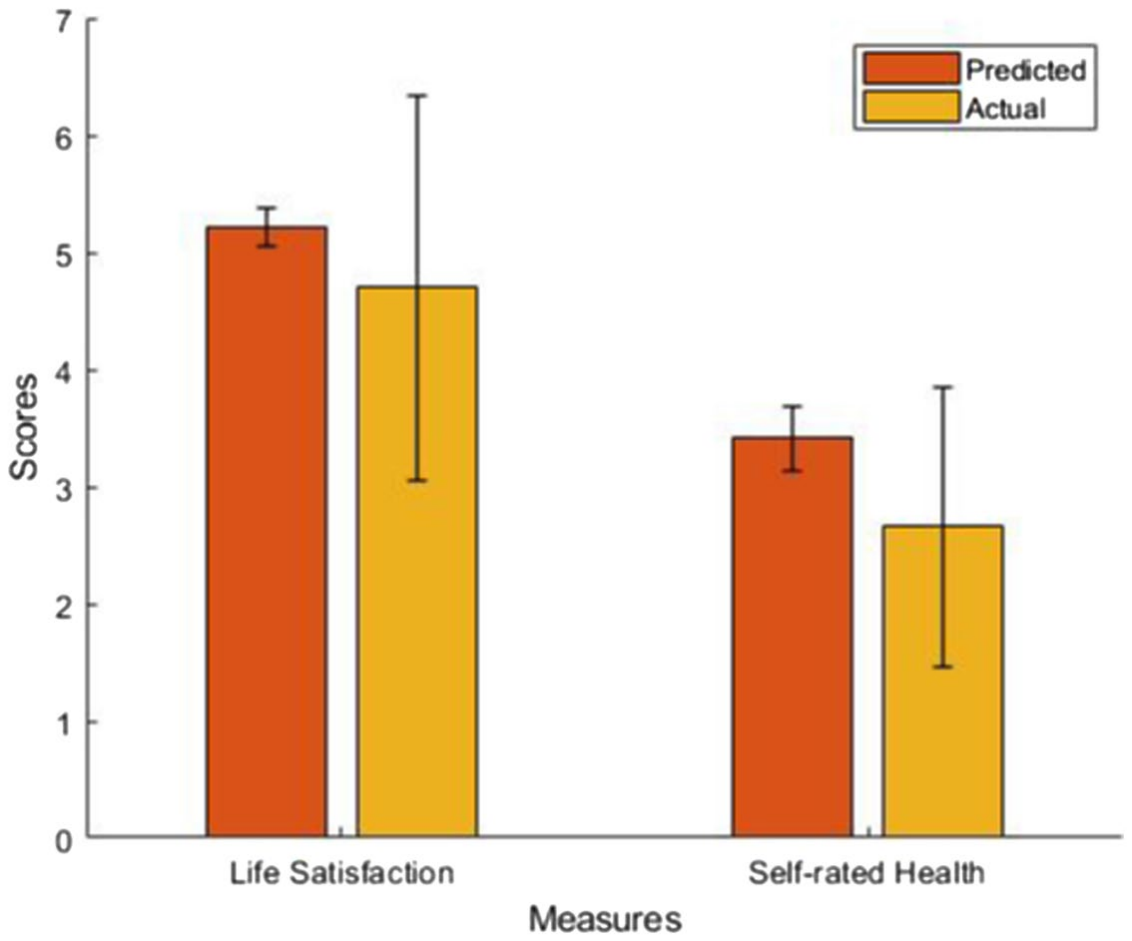
The predicted and actual score of life satisfaction and SRH in people with epilepsy given their demographics based on the generalized linear models trained on people without epilepsy.

The estimates of the demographics trained in people without epilepsy can be found in Table 2. The main findings of the current study were that people with epilepsy have poorer life satisfaction (*t*(427) = −6.59, *p* < 0.001, Cohen’s *d* = −0.32, 95% C.I. [−0.68, −0.37]) and poorer SRH (*t*(427) = −13.83, *p* < 0.001, Cohen’s *d* = −0.67, 95% C.I. [−0.87, −0.65]) compared to the predicted scores. The mean and standard deviation of predicted and actual scores were plotted in Figure 1.

**Table 2:** The estimates (*b*) of linear models trained based on demographic predictors of people without epilepsy.

	Life satisfaction	SRH
Age	0.00 ***	−0.02 ***
Sex	0.07 ***	0.01
Monthly income	0.00 ***	0.00 ***
Highest educational qualification	0.07 ***	0.30 ***
Legal marital status	0.25 ***	0.17 ***

*p < 0.05; **p < 0.01; ***p < 0.001.

## Discussion

The aim of the current study is to investigate how epilepsy is related to life satisfaction and SRH in people with epilepsy from the United Kingdom. By using an innovative train-and-test approach that takes demographic characteristics into account. The results showed that people with epilepsy have both poorer life satisfaction and SRH compared to the scores that would be predicted by their demographics.

The current findings were largely consistent with the literature regarding epilepsy as negatively related to the quality of life and life satisfaction (Leidy et al., 1999; Kobau et al., 2008, 2012; Boling et al., 2018) and SRH (e.g., Kobau et al., 2007, 2008). Indeed, people with epilepsy face a lot of challenges in their daily life (Leidy et al., 1999; Centers for Disease Control and Prevention, 2005; Kobau et al., 2008, 2012; Layne Moore et al., 2009; Trinka et al., 2019). Second, perceived limitations in social and emotional support can also lead to poor life satisfaction (Kobau et al., 2008; Malvaso and Kang, 2022) and SRH. Third, neurological factors have adverse impacts on the quality of life in people with epilepsy (Hermann and Jacoby, 2009) as well. These challenges may in turn explain the poorer life satisfaction and SRH in the current study.

However, the level of seizure control, the type of treatment, and the presence of comorbid mental illness were not controlled in the current sample. For instance, Kobau et al. (2006) found that adults with epilepsy are more likely to have experienced depression or anxiety during the last year. Moreover, long-term mood and life satisfaction are strongly correlated and are a stronger predictor of the current mood (Pavot and Diener, 1993). As pointed out by Kobau et al. (2007), it is certainly possible that chronic depressive disorder may explain the associations found in the current study, especially the association between epilepsy and SRH. Thus, these findings emphasized the need to assess seizure control, adverse treatment side effects, and untreated disease comorbidity (e.g., mental illness) that might explain the poor life satisfaction SRH in people with epilepsy. Moreover, 84% of adults with epilepsy comorbid with psychological distress had a need for mental health care in the past year, but only 57% came to see a mental health provider in that time (Thompson et al., 2012). There were also about 27% of people with active epilepsy and seizures reported that they did not see a neurologist or epilepsy specialist in the past year, which suggested a treatment gap in appropriate epilepsy care (Kobau et al., 2008; Thompson et al., 2012) that can have a negative impact on actual and perceived health status. In addition, self-reported measures and the cross-sectional design of the current study were important limitations of the current study.

To conclude, the current study looked at how life satisfaction and SRH are affected by epilepsy by using an innovative train-and-test approach that could well control demographics. The current findings suggested that people with epilepsy have poorer life satisfaction and SRH compared to people without epilepsy, which may imply that both life satisfaction and SRH are valid measures of wellbeing in people with epilepsy. These findings may indicate that improving life satisfaction and SRH is critical in people with epilepsy. Healthcare professionals may utilize findings from the current study to come up with ways that can benefit wellbeing of people with epilepsy. Specifically, there are several ways to achieve this, including social participation (Ichida et al., 2013), e-Health tools (Seiwert et al., 2022), healthy health diets (Abuladze et al., 2017) can improve SRH and life satisfaction in people with epilepsy.

## Data availability statement

Publicly available datasets were analyzed in this study. This data can be found at: https://www.understandingsociety.ac.uk.

## Author contributions

WK: conceptualization, data curation, formal analysis, investigation, methodology, resources, software, supervision, writing—original draft, and writing—review and editing.

## Funding

This work was supported by the Imperial Open Access Fund.

## Conflict of interest

The author declares that the research was conducted in the absence of any commercial or financial relationships that could be construed as a potential conflict of interest.

## Publisher’s note

All claims expressed in this article are solely those of the authors and do not necessarily represent those of their affiliated organizations, or those of the publisher, the editors and the reviewers. Any product that may be evaluated in this article, or claim that may be made by its manufacturer, is not guaranteed or endorsed by the publisher.
